# Updating prospective marriage and divorce data on Norwegian cohorts of two-sex marriages from 1886 until 2018 with same-sex marriages from 1993 until 2018

**DOI:** 10.1016/j.dib.2024.110584

**Published:** 2024-06-03

**Authors:** Rune Zahl-Olsen

**Affiliations:** Department of child and adolescent mental health, Sørlandet Hospital, Kristiansand, Norway

**Keywords:** Same-sex, Homosexuality, Marriage, Divorce, Prospective, Follow-up, Urban, Rural

## Abstract

This paper presents an update to the previously published dataset known as prospective marriage and divorce data on Norwegian cohorts of two-sex marriages from 1886 until 2018. This update adds prospective data from all same-sex marriages formed in Norway between 1993 and 2018, with annual follow-up for 25 years, totaling 26 cohorts and 5,187 marriages. The data list the number of marriages that ended in divorce throughout each year of follow-up. The data contain information about the age of both spouses, the number of divorces from each cohort in the total population of marriages, as well as divorces among marriages formed in urban and rural areas of the country. Marriages formed within a calendar year are pooled into cohorts, and each pair is examined annually to ensure that the same two people remain married. As a result, the method is equivalent to the initial dataset on two-sex marriages presented in the first dataset.

Specifications Table

**Table:** 

Subject	*Same as in original data article*
Specific subject area	*Marriage and divorce among same-sex marriages*
Type of data	*Same as in original data article*
How the data were acquired	*Same as in original data article*
Data format	*Same as in original data article* Raw
Description of data collection	One statistician at Statistics Norway, who had access to information on every person and their marital status, created the dataset. This update includes all same-sex marriages formed in Norway from the beginning of 1993 until the end of 2018. Due to Norwegian legislation, same-sex marriages from 1993 until 2008 were officially registered partnerships, and only in 2009 were same-sex marriages. When the legislation changed in 2009, all same-sex marriages became same-sex marriages. This dataset does not make this distinction. The data does not differentiate between individuals who have been previously married and those who have not.
Data source location	Institution: Statistics Norway Country: Norway The major data source comprises information about every individual in Norway on a variety of characteristics, and hence contains sensitive information. Statistics Norway makes primary data available only to certified employers. However, researchers connected with a research institution certified by The Research Council of Norway or Eurostat may obtain microdata on a case-by-case basis.
Data accessibility	Repository name: Mendeley Data Data identification number: DOI:10.17632/jx73wpcx2c.1 And DOI:https://doi.org/10.17632/cr8thpfmvm.2 Direct URL to data [5,6]: https://data.mendeley.com/datasets/jx73wpcx2c/1 and https://data.mendeley.com/datasets/cr8thpfmvm/2
Related data article	Zahl-Olsen, Prospective marriage and divorce data on Norwegian cohorts of two-sex marriages from 1886 until 2018, (2022), Data in Brief. https://doi.org/10.1016/j.dib.2022.108083
Related research article	R. Zahl-Olsen, F. Thuen, Same-sex Marriage over 26 years: Marriage and Divorce Trends in Rural and Urban Norway, Journal of Family History. (2022). https://doi.org/10.1177/036319902211229

## 1 Value of the Data

• Over 30 countries worldwide have legalized same-sex marriages, but the previous data did not include any information on this population.
• Male and female same-sex marriages differ in a number of ways, and this dataset contains statistics on Norway's total population from the first year that same-sex marriages were legalized. The data are of significant practical value since they can be utilized to make cross-cultural comparisons between male and female same-sex marriages in rural and urban locations and two-sex marriages

## 2 Data Description

This data is an update of a previous dataset containing information on two-sex marriages in Norway [1] by adding data on same-sex marriages. The data set adds a 26-year time series of same-sex marriage and divorce statistics. Since 1993, Norway has legalized same-sex registered partnerships and permitted same-sex weddings in 2009. Over time, the age at which couples enter marriage and the number of married individuals have varied. The data is organized in two Excel and CSV files.

**Fig. 1:**
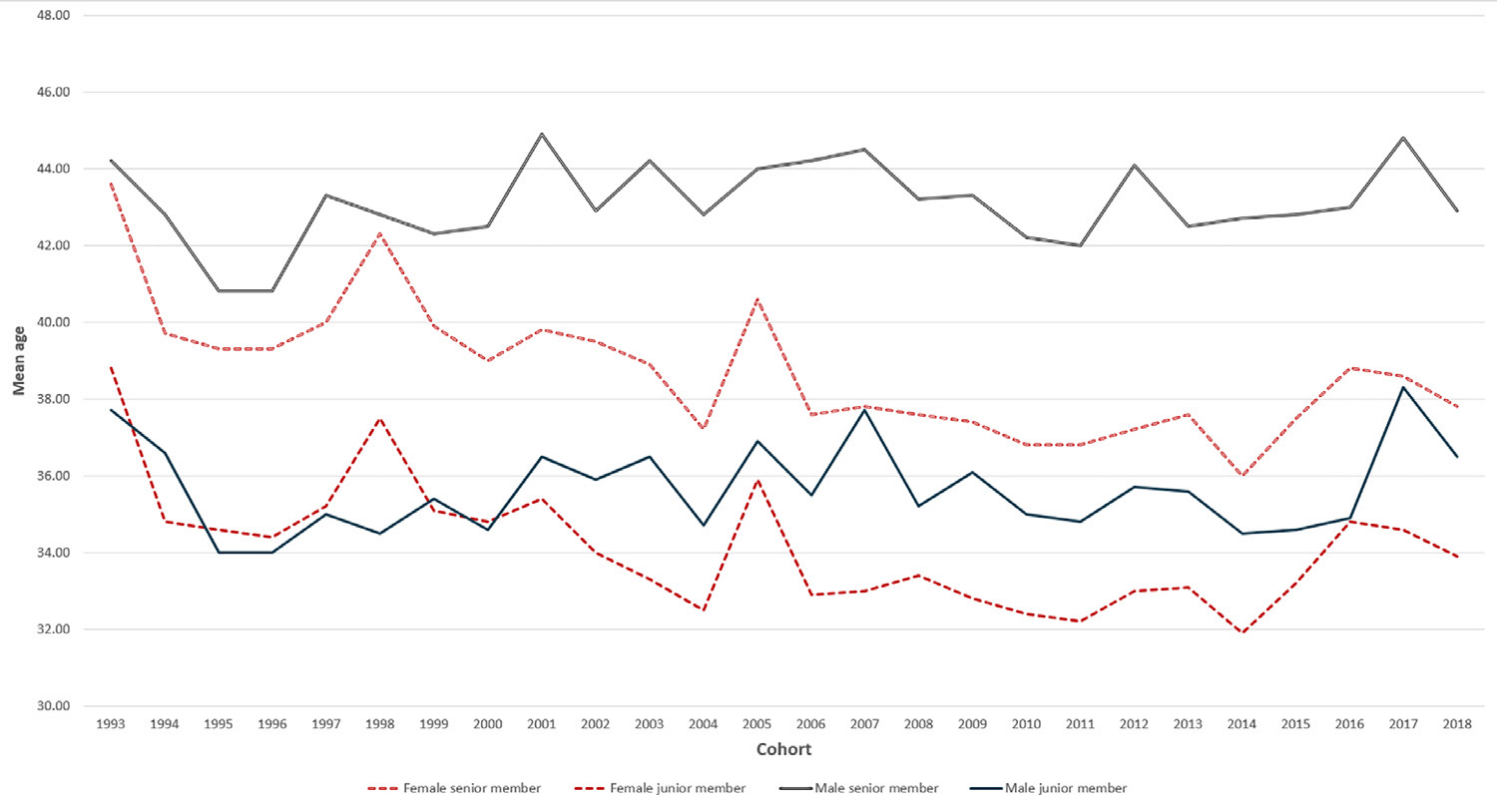
Mean age at marriage for senior and junior members of same-sex couples in each cohort.

The first file, “Same-sex_age,” presents the age of the senior and junior members of a couple. The file has seven columns in which the first three variables determine the sample. The data contains three samples: the total population in Norway as well as marriages formed in urban and rural areas. Weddings were categorized as urban or rural based on the population size of the municipality where the oldest person involved in the wedding was officially registered. In 2020, Norway had a total of 356 municipalities. The population of these municipalities was ordered in descending order, from the largest to the lowest. The urban sample consisted of marriages from the six most populous municipalities, encompassing the five largest cities. The rural sample excluded marriages from 27 municipalities with a moderate population size and included marriages from the remaining 323 small communities. The urban group consisted of municipalities with a population exceeding 110,000, while the rural group consisted of municipalities with a population below 31,000. The definition of urban and rural areas in Norway is the same as in the previous data presented by Zahl-Olsen [1] that was used in a study of two-sex marriages [2]. A study on same-sex marriages [3] used the data from this study. Fig. 1 visualize the distribution of mean age at marriage of the senior and junior members of male and female same-sex couples for each of the included cohorts.

Sample: urban, rural, and total
Category: 2 = same-sex women, and 3 = same-sex men
Cohort: 1993–2018.
Age1: The mean age of the couple's senior member for that sample.
SD1: Standard deviation of the couple's senior member for that sample.
Age2: The mean age of the couple's junior member for that sample.
SD2: Standard deviation of the couple's junior member for that sample.

The second file, “Same-sex_Divorce_rate,” presents data on divorce rates for male and female same-sex marriages for every year of follow-up for each sample of the cohorts included in the study. The data is structured in the same manner as in the first file, and the first three variables, sample, category, and cohort, define the sample. The following variables describe the number of marriages and divorces for each of the samples.

Population is the number of marriages formed in that cohort of the specified sample, e.g., the number of male same-sex marriages in urban areas of Norway in 2010.

Follow_up_year is the year of follow-up (0–25). Each couple was investigated for each year to see if the same two people were still married to each other.

Accum_N_Divorced is the accumulated number of divorces in the selected sample, category, and cohort at that year of follow-up.
Accum_divorced is the Accum_N_Divorced divided by population (the initial number of married people in that cohort).
Year_divorced is the value in the cohort variable added to the value of the follow-up_year variable.

## 3 Experimental Design, Materials and Methods

Statistics Norway's database has a unique identifying number for each resident of Norway. If a person marries, this information is associated with their unique ID. If the individual undergoes divorce, this will also be recorded and associated with that number. We used this information about individuals to monitor married couples to guarantee that they were not just married each year, but also to the same person each year. This method allowed us to trace each marriage and identify any divorces, even if a person divorced and remarried in the same year. We included unions in which at least one spouse resides in Norway. Some argue that separation reports are preferable to divorce reports when evaluating divorces [4], but separation is a reversible status that is not always recorded in the Norwegian database. In Norway, divorcing couples typically separate before filing for divorce. People who are separated are counted as married in this data because they are able to withdraw from their separation.

## Ethics Statement

Participant data were fully anonymized and provided in compliance to data redistribution policies from Statistics Norway.

## Declaration of Generative AI and AI-Assisted Technologies in the Writing Process

During the preparation of this work, the author used QuillBot.com in order to improve the language since the author's first language is not English. After using this tool/service, the author reviewed and edited the content as needed and take full responsibility for the content of the publication.

## CRediT authorship contribution statement

**Rune Zahl-Olsen:** Conceptualization, Methodology, Writing – original draft.

## Data Availability

Dataset of urban and rural same-sex marriages and divorces in Norway 1993 to 2018 (Original data) (Mendeley Data).
